# Prevalence and factors associated with postpartum pelvic girdle pain among women in Poland: a prospective, observational study

**DOI:** 10.1186/s12891-022-05864-y

**Published:** 2022-10-20

**Authors:** Małgorzata Starzec-Proserpio, Maria Węgrzynowska, Dorota Sys, Anna Kajdy, Witold Rongies, Barbara Baranowska

**Affiliations:** 1 Department of Midwifery, Centre of Postgraduate Medical Education, ul. Żelazna 90, 01-004 Warsaw, Poland; 2 Department of Reproductive Health, Centre of Postgraduate Medical Education, ul. Żelazna 90, 01-004 Warsaw, Poland; 3 Department of Rehabilitation, Faculty of Medical Sciences, Medical University of Warsaw, ul. Ks. Trojdena 2C, 02-109 Warsaw, Poland; 4 Ist Clinic of Obstetrics and Gynecology, Centre of Postgraduate Medical Education , ul. Żelazna 90 , 01-004 Warsaw, Poland

**Keywords:** Pelvic girdle pain, Postpartum period, Prevalence, Pelvic floor, Pelvic floor disorders, rectus abdominis

## Abstract

**Background:**

Pelvic girdle pain (PGP) is a type of pregnancy-related lumbopelvic pain. This study aimed to examine the prevalence, severity, and factors associated with postpartum PGP in a selected group of postpartum women in Poland.

**Methods:**

This was a prospective, observational study. In phase 1, 411 women were recruited 24–72 h postpartum. The prevalence of PGP was assessed by a physiotherapist using a series of dedicated tests. Pelvic floor muscle function and presence of diastasis recti were assessed via palpation examination. Age, education, parity, mode of delivery, infant body mass, body mass gain during pregnancy, the use of anesthesia during delivery and were recorded. In a phase 2, 6 weeks postpartum, the prevalence of PGP and its severity were assessed via a self-report.

**Results:**

In phase 1 (shortly postpartum), PGP was diagnosed in 9% (n = 37) of women. In phase 2 (6 weeks postpartum), PGP was reported by 15.70% of women (n = 42). The univariable analyses showed a higher likelihood of PGP shortly postpartum in women who declared PGP during pregnancy (OR 14.67, 95% CI 4.43–48.61) and among women with abdominal midline doming (OR 2.05, 95% CI 1.04–4.06). The multivariable regression analysis showed significant associations in women with increased age (OR 1.12, 95% CI 1.01–1.21) and declaring PGP during pregnancy (OR 14.83, 95% CI 4.34–48.72).

**Conclusion:**

Although the prevalence of postpartum PGP among women in Poland is lower than reported in other countries, it is experienced by almost every tenth women shortly postpartum and every sixth can report similar symptoms 6 weeks later. Age, PGP during pregnancy and abdominal midline doming were associated with experiencing PGP shortly postpartum.

## Background

Symptoms of pelvic girdle pain (PGP) are commonly reported to healthcare providers by pregnant or postpartum women. This musculoskeletal disorder is a form of low back pain experienced between the posterior iliac crests and the lower edge of the gluteal folds, most commonly in the vicinity of the sacroiliac joints. PGP also includes pain in the pubic symphysis, occurring in isolation or in conjunction with other pelvic joints. Patients with PGP have reduced tolerance to standing, walking, sitting, and changing positions [1]. Pregnancy-related PGP may appear as early as the first trimester of pregnancy or can be delayed up to 3 weeks postpartum [2]. PGP is associated with significantly more pain and functional limitations than lower back pain [3]. The pain often subsides after delivery, but some women continue to have persistent symptoms postpartum. Among women reporting PGP during pregnancy, 1 in 10 will suffer from it up to 11 years later [4]. This significantly impacts their quality of life [5]. In Poland, the prevalence of PGP during pregnancy was reported by 42% of women [6]. To our knowledge, there are no studies reporting on the prevalence of postpartum PGP in Poland or any other country in the central-eastern region of Europe.

Assessment of muscle impairments has been recommended in Clinical Practice Guidelines for PGP in the postpartum population [7]. To date, several studies investigated musculoskeletal factors that could be associated with pregnancy-related PGP, including the function of pelvic floor muscles and diastasis rectus abdominis (DRA). Coordination between lumbopelvic and abdominal muscles and fascia was suggested to play a significant role in postural stabilization [8]. It was hypothesized that insufficient motor control could give rise to pain from impaired load transfer throughout the pelvic girdle [9], and that pelvic floor and abdominal muscles play an important role in the stabilization and motor control of the pelvis [8, 10]. However, there is still little evidence to support these associations. While some studies confirm the relationship between the DRA, linea alba dysfunction, and postpartum PGP [11, 12], others do not [13–15]. A recent systematic review concluded that DRA presence might be associated with decreased abdominal muscle strength and severity of low back pain and suggested further studies rigorously assessing this association [16]. A literature review investigating the relationship between perineal characteristics and PGP suggested that overactivity and increased tension of pelvic floor muscles are more common in women with PGP [17].

This study aimed to assess the prevalence and severity of PGP among women in Poland early postpartum and 6 weeks after delivery. Additionally, we aimed to identify factors associated with early postpartum PGP, including also DRA and pelvic floor function.

## Methods

This was a prospective, observational study. Ethics approval was received from the Bioethics Committee of the Medical University of Warsaw (KB/136/2017) and the study was registered at https://www.anzctr.org.au/ under the number ACTRN 12,618,000,764,235. Data was collected between 1.12.2017 and 12.03.2020. All participants provided written informed consent prior to commencing any of the study procedures. The study was supported by the Department of Midwifery at the Centre of Postgraduate Medical Education Research Program for 2020. The STROBE checklist was followed to ensure proper reporting of this study [18].

### Setting

St. Sophia Specialist Hospital in Warsaw, Poland served as a recruitment site for this study. It is a tertiary, publicly funded hospital with over 6500 births annually. A free consultation with a pelvic health physiotherapist is part of standard care for every woman after delivery at this hospital. The participants were recruited from among the women attending the consultation.

### Participants

Women between 18 and 45 years old who attended free physiotherapy consultation 24–72 h postpartum were invited to participate in the study. The main exclusion criteria were additional comorbidities potentially causing PGP-like symptoms (rheumatoid arthritis, ankylosing spondylitis, Scheuermann disease, Ehlers-Danlos syndrome, spinal surgeries, nerve root compression, spondylolisthesis), contraindications for pelvic floor examination (puerperal genital hematoma, diffuse perineal edema, perineal wound dehiscence, bladder catheterization) and severe postpartum complications (internal bleeding, femoral artery embolism, pelvic fracture). To limit the sampling bias and ensure that patients were randomly included in the study, every third participant of the postpartum physiotherapy consultation was invited, and recruitment for the study took place every third day.

### Procedure

The study was conducted in two phases. Phase 1 was carried out at the hospital. Participants who met the inclusion criteria and gave informed consent were included in the study. Age, education, parity (defined as previous deliveries > 24 weeks gestation), delivery type (vaginal, forceps/vacuum extractor, cesarean), infant body mass (< 4000 g/4000 g or more), height, body mass gain during pregnancy, the use of anesthesia during delivery were recorded from the patient medical record and confirmed via self-report. The women were asked if they had experienced PGP during the last pregnancy (yes/no and if yes - in what location) and urinary incontinence during or before the pregnancy (yes/no). The presence and severity of PGP were assessed by the physiotherapist. Additionally, each woman received an examination of the pelvic floor and abdominal muscles. All examinations were performed by the same registered pelvic health physiotherapist who had completed advanced training in urogynaecology and was certified by the Polish Urogynecological Society to digitally examine the pelvic floor muscles.

In phase 2 (“follow-up”), all women who participated in phase 1 were contacted via text message 6 weeks postpartum. In the message, they were asked if they experienced PGP. In case of no response within 48 h, another message was sent. No response to the second message meant a loss to follow-up.

### Measures/Variables

#### PGP prevalence and severity

**Fig. 1:**
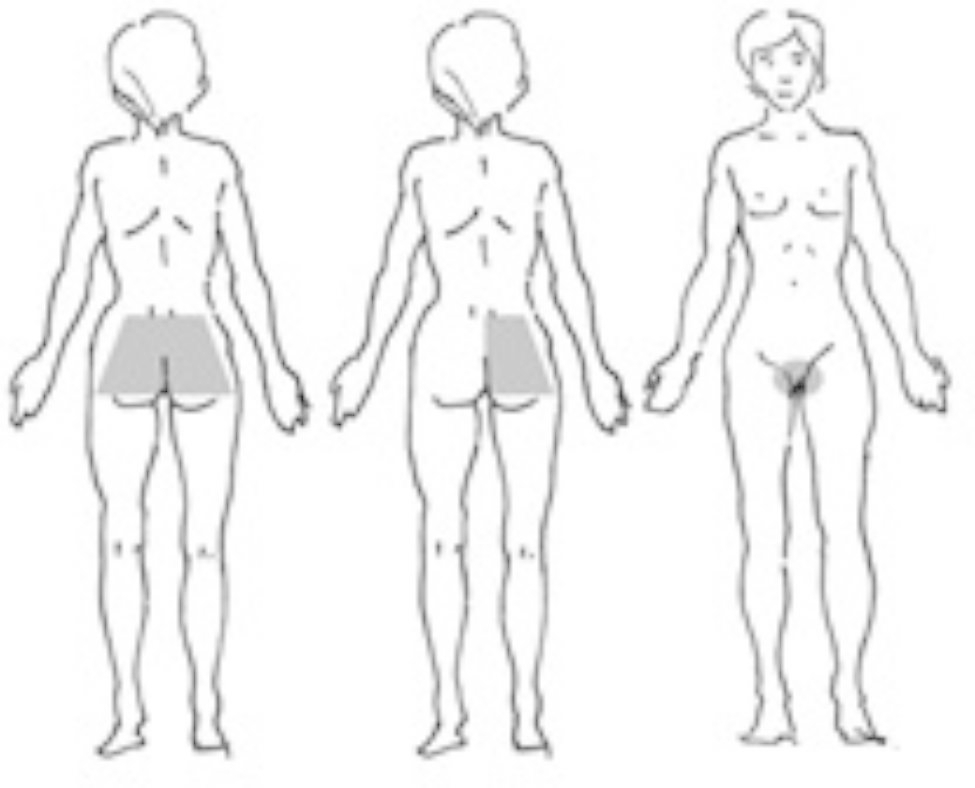
Pain maps used for assessing the presence of PGP

During the examination by the physiotherapist in phase 1 (shortly postpartum), every patient was asked the question: “Do you have pelvic girdle pain in the places marked in the figure (Fig. 1), which aggravates during activities such as standing up, walking, or rolling from side to side?“ The participants reported their pain by indicating its location on a body chart. This was confirmed by pointing to the site of the pain in their body. The physiotherapist then carried out a further clinical examination to confirm the presence of PGP. For PGP classification, existing guidelines and previous reports were used [1, 3, 4]. Firstly, the lumbar spine examination was performed (flexion/extension movements, lateral rotations, lateral bends, Laseque test) to exclude lumbar causes of pain in the pelvic girdle region. This was followed by the tests dedicated to the pelvic girdle: Posterior Pelvic Pain Provocation (P4) test, distraction test, compression test, palpation of the pubic symphysis, modified Trendelenburg test, active straight leg raise test (ASLR). At least two tests had to be positive for PGP to be confirmed. For the ASLR test, the scores on both sides were added, and the total score ranged from 0 to 10. An ASLR total score of 4 and above was considered positive.

Patients with confirmed PGP rated their mean pain intensity during a day on a Numerical Rating Scale (NRS) from 0 to 10, where 0 meant no pain and 10 meant the worst pain imaginable [19]. We designated the categorical cut-off points for the NRS as mild (1–4), moderate (5–6), and severe (7 to 10) [20]. Functional limitations were assessed with the Polish version of The Pelvic Girdle Questionnaire (PGQ) [21]. Values 0–28 were interpreted as low, 28–62 as moderate, and > 62 as high. [22]

Assessment of PGP prevalence 6 weeks postpartum was carried out via text message. To increase the certainty of our results, we have provided the figure with marked pain locations, and also a short description of PGP-related symptoms. The women were asked: “Are you currently experiencing pelvic/sacrum/coccyx/pubic symphysis pain (Fig. 1) with or without a feeling of instability in these areas, arising or worsening with changes in position or movement?”. The message was accompanied by an image with marked pain locations (Fig. 1). Women were classified as having pelvic girdle syndrome if they indicated 1st and 3rd location. If the response was positive, the patient was asked to rate the pain using NRS and functional limitations by filling out and returning the PGQ via email or MMS.

#### Pelvic floor measurements

A pelvic floor assessment was performed using the palpation examination. We decided this would be the only appropriate method to be used in the early postpartum period as it is fast, painless, non-invasive, and requires no additional equipment. All patients were assessed in a crook lying position. The PERFECT scheme [23] with the 6-point OXFORD scale (0–5) was used to evaluate a maximal voluntary contraction (MVC) of the pelvic floor. The OXFORD scale is a reliable measure of the MVC with acceptable intra-observer and test-retest reliability [24]. The existing research showed that the palpation assessment using the OXFORD scale was consistent with the ultrasound examination results [25].

A seven-point scale proposed by Reising et al. was used to assess muscle tone with values ranging from − 3 (very hypotonic muscles) to + 3 (very hypertonic muscles) with 0 stating normal tone. This scale has been studied for its reliability showing fair-to-moderate interrater reliability with correlation coefficients of 0.2–0.5 [26]. Weak-to-fair associations between the Reissing scale, dynamometry, and ultrasound imaging with correlation coefficients of 0.2–0.4 have been previously shown [27].

Additionally, possible activation of synergistic muscles (gluteal muscles, adductors, and abdomen), and the ability to activate the pelvic floor muscles without breath-holding were observed during the pelvic floor examination. That was done in order to determine the correctness of pelvic floor activation: isolated pelvic floor contraction without breath-holding was considered correct.

#### DRA measurements

**Fig. 2:**
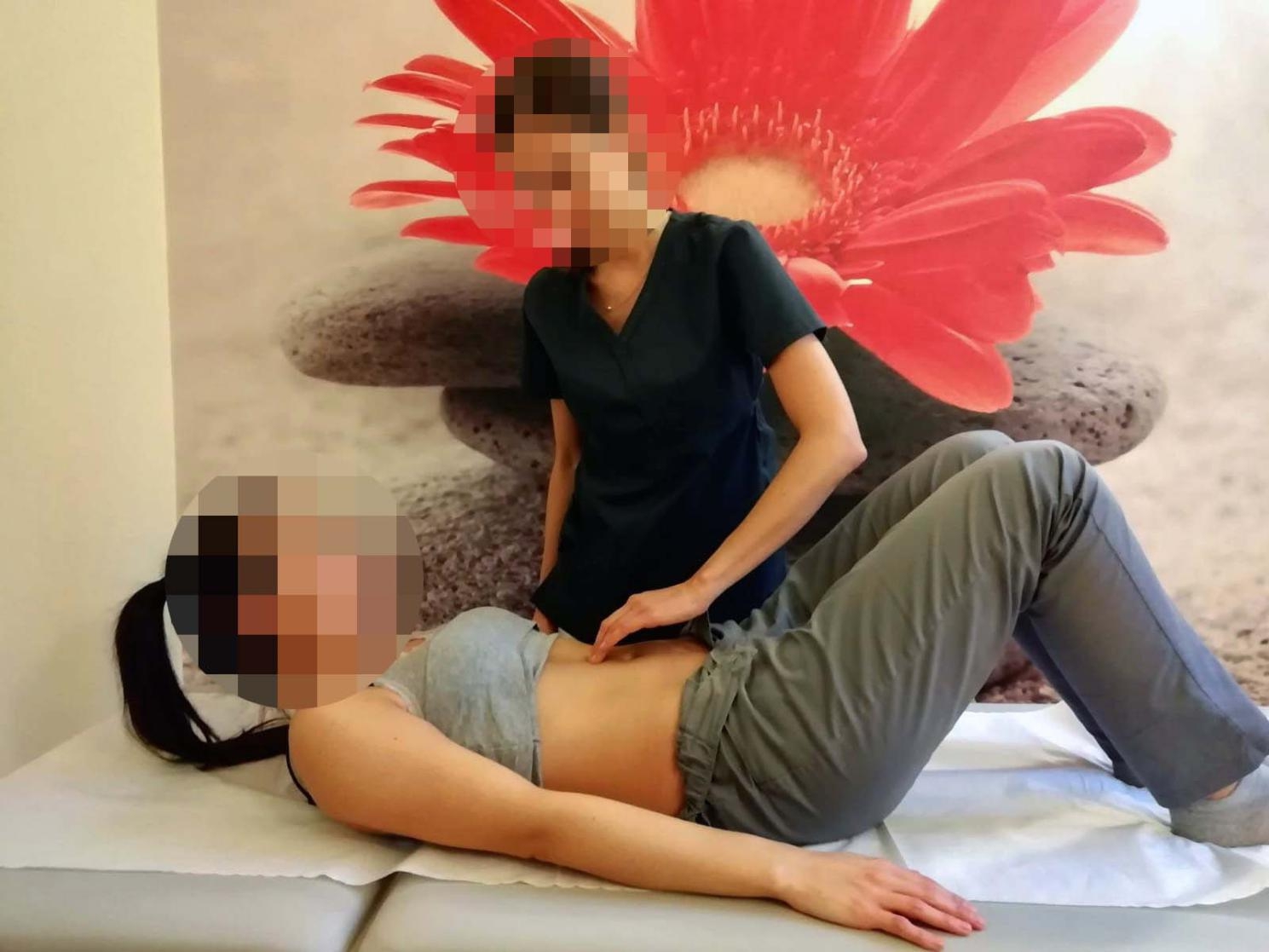
DRA and linea alba assessment

The width of rectus abdominus muscle bellies (inter-recti distance, IRD) was determined by palpation using the procedure reported in previous studies [28]. The women were asked to lie in a standard supine position with their knees bent. Then, they were asked to perform the abdominal curl-up by raising the head and upper torso until the shoulder blades left the examination bed (Fig. 2). Measurements were taken at the navel level and 4.5 cm above and below it [29, 30]. As done in previous studies, the point of largest width was selected for the analysis [13–15]. Mota et al. showed good intra-rater reliability (K_w_>0.7) in terms of palpation measurements of IRD [28] and Benjamin et al. showed moderate to very good correlation of IRD palpation with ultrasound (r = 0.75–0.98) [31]. According to other studies implementing this method [13, 15, 29, 30] DRA was considered when the IRD value was ≥ 2 fingerbreadths. The participants in this study were divided into four categories depending on the largest palpation measurement (number of fingers) in one of three locations: no DRA (IRD < 2 fingerbreadths), mild DRA (IRD 2;< 3), moderate DR (IRD 3;<4), severe DRA (IRD > 4) [13].

It has been suggested that the integrity of linea alba may influence the capacity to stabilize the pelvis and the lumbar spine, and the ability of the linea alba to transmit forces across the midline may have a more significant impact on function than the magnitude of the IRD [32]. Therefore, in the context of the assessment of DRA and linea alba dysfunction, not only IRD but also the stiffness and distortion/bulging at the level of linea alba could play an important role [32, 33]. To investigate any possible association with PGP, we have adopted a simplified method of assessing the doming of the abdominal midline. It was defined as “abdominal midline doming” (yes/no). During the curl-up test, the physiotherapist observed whether the abdominal midline bulged. Although this method does not allow to determine which structures are bulging (e.g., linea alba or “just” subcutaneous tissue), it may give us a simplified estimation of pressure management in the abdominal cavity. A similar assessment has recently been used in another research [34].

### Statistical analysis

The sample size was determined based on a priori calculations, based on the reports of Wu et al., in which the incidence of postpartum low back pain and /or PGP is estimated at 25% [35]. The following formula was used:

$$n=\frac{{Z}^{2}P(1-P)}{{d}^{2}},$$

where n is the sample size, the Z value (corresponding to the significance level p 0.05) is 1.96. P value is the expected occurrence of pain, and the d value is 0.05 (recommended value for an expected prevalence between 10 and 90% [36]). Based on this calculation, the minimum sample size was 288. A high drop-out rate was also expected due to the specificity of the postpartum period, as observed by other authors [37]. To secure the minimal number of participants for phase 2 of the study (“follow-up”), the sample size was increased to a minimum of 400.

For continuous variables, mean and SD were calculated. Categorical data are presented as numbers and percentages. The prevalence of PGP was calculated by dividing the number of women classified with PGP by the total number of women who participated in the study. To assess the possible differences between women who responded 6 weeks postpartum and those who were lost to follow-up, Student *t-test*, the Mann-Whitney *U* and the the χ^2^ test were used depending on the type and normality of data.

Univariable logistic regression analyses were fitted to test individual factors for association with PGP shortly postpartum (phase 1). Multivariable logistic models were used to identify associations with more than one factor included in the model. The selection of factors to include in the multivariable models was informed by the univariable results. Variables with p-values less than or equal to 0.1 were included in the multivariable regression model. The number of possible investigated factors in the multivariable model was calculated assuming 10 participants per potential associated factor with division by the obtained prevalence rate [38]. The best subset of explanatory variables was selected manually by excluding the variables with the smallest contribution to the model. The predictive power of the model was calculated by Nagelkerke R-square (R²). Missing data were not included in the analysis. Alpha was set at 0.05. Statistical analyses were performed using PQStat ver. 1.8.2.166.

## Results

A total of 415 women were invited to participate, 4 of whom did not consent to join the study. Thus, 411 women were included in phase 1 (shortly postpartum). Table 1. presents the characteristics of the participants. In phase 2 (“follow-up”), 268 women replied to the text message (65.2% of the initial group). No statistically significant differences were found between those who responded to the message and those who did not in terms of variables assessed shortly postpartum (in phase 1): BMI, age, presence of PGP postpartum, parity, DRA severity, and ability to activate pelvic floor muscles.

**Table 1:** Characteristics of the study group, n = 411

	All participants n = 411	With PGP n = 37	Without PGP n = 374
**Age**	31.17 ± 4.01	32.35 ± 4.33	31.06 ± 3.96
**Height [cm]**,	167.46 ± 5.69	167.00 ± 6.64	167.51 ± 5.60
**BMI before pregnancy**	22.14 ± 3.57	22.46 ± 3.20	22.10 ± 3.60
**Body mass gain in pregnancy [kg]**	14.30 ± 4.94	14.92 ± 4.28	14.24 ± 4.99
**Education, n (%)** †			
vocational education	2 (0.5)	1	1
secondary education	43 (10.5)	2	42
university education	366 (89)	35	331
**Parity, n (%)**	1.72 ± 0.96	1.95 ± 1.10	1.70 ± 0.94
1	209 (50.80)		
2	143 (34.80)		
3	37 (9.00)		
4	12 (3.00)		
5	8 (2.00)		
6	1 (0.20)		
7	1 (0.20)		
**Mode of the last delivery, n (%)**			
vaginal	387 (94.20)	37 (100)	350 (93.60)
cesarean ‡	18 (4.30)	0	18 (4.80)
vacuum extractor ‡	6 (1.50)	0	6 (1.60)
**Perineal injury during recent delivery, n (%)**			
none	162 (39.50)	18 (48.60)	144 (38.50)
1st grade	138 (33.50)	9 (24.30)	129 (34.50)
episiotomy	111 (27)	10 (27)	101 (27)
**Anesthesia during last delivery, n (%)**			
none	238 (57.91)	24 (64.86)	214 (57.22)
epidural	154 (37.47)	12 (32.43)	142 (37.97)
spinal	19 (4.62)	1 (2.70)	18 (4.81)
**Infant body mass ≥ 4000 g**			
yes	336 (81.75)	6 (16.22)	330 (88.24)
no	75 (18.25)	31 (83.78)	44 (11.76)

† Participants with vocational education were excluded (from this calculations) because of the low number of records; ‡ no statistical analysis due to low number of observations; χ² Chi2 tet value; U Manna Whitney test value

Among the patients in phase 1 of the study (shortly postpartum), 47.9% (n = 197) reported PGP symptoms during pregnancy. In phase 1, PGP was diagnosed in 9.0% (n = 37) of women at the early postpartum stage. In phase 2 (6 weeks postpartum), PGP was reported by 15.7% of women (n = 42). Table 2 presents the prevalence and type of PGP across different time points, and Table 3 shows the severity of pain and functional disturbances in both phases of the study.

**Table 2:** Prevalence and types of PGP across the time points.

Timepoint		During pregnancy		Early postpartum		6 weeks postpartum	
**Assessment form**		**self-reported, retrospective**		**clinical assessment**		**self-reported**	
		**N**	**%**	**N**	**%**	**N**	**%**
**PGP**		197/411	47.93	37/411	9	42/268	15.70
**PGP** **type**	**Posterior Pelvic Pain**	86/197	43.70	9/37	24.30	16/42	38.10
	**Unilateral pain**	17/197	8.60	2/37	5.40	2/42	4.80
	**Symphyseal pain**	44/197	22.30	9/37	24.30	12/42	28.60
	**Pelvic Girdle Syndrome**	47/197	23.90	16/37	43.20	11/42	26.20
	**Unilateral pain + symphyseal pain**	3/197	1.50	1/37	2.70	1/42	2.40

**Table 3:** The severity of pain and functional limitations at phase 1 and 2 of the study

Timepoint	Early Postpartum		6 weeks postpartum	
	**Mean (SD)** **(min-max)**	**N**	**Mean (SD)** **(min-max)**	**N**
**NRS**	5.34 (1.74) (3–9)	37/37	4.63 (2.04) (1–8)	24/42
**PGQ [%]**	48.87 (16.40) (23.61–82.61)	32/37	25.80 (14.90) (6.67–56.94)	13/42

The values of the PGQ questionnaire from the early postpartum stage were missing in five cases – they were left blank or only partially completed. Six weeks after delivery, the pain intensity value on the NRS was reported by 24 women, and the PGQ questionnaire was completed only by 13

The univariable analyses showed a higher likelihood of PGP shortly postpartum in women who declared PGP during pregnancy and among women with doming at the abdominal midline in the projection of linea alba (Table 4.).

**Table 4:** Univariable analysis of factors associated with pelvic girdle pain (PGP) shortly postpartum (phase 1)

		PGP (+) n = 37	PGP (-) N = 374	OR (95% CI)	p-value
**PGP during pregnancy (yes)** **n (%)**		34 (91.90)	163 (43.60)	14.67 (4.43–48.61)	**< 0.01**
**Age** **mean (SD)**		32.35(4.33)	31.06 (3.96)	1.08 (0.99–1.18)	0.06
**Body mass gain during pregnancy** **mean (SD)**		32.68 (4.60)	31.59 (4.37)	1.03 (0.96–1.10)	0.42
**BMI before pregnancy** **mean (SD)**		22.46 (3.20)	22.1 (3.60)	1.03 (0.94–1.12)	0.56
**Number of previous deliveries mean (SD)**		1.95 (1.10)	1.7 (0.94)	1.27 (0.93–1.71)	0.14
**Urinary incontinence during pregnancy or before** **(yes) n (%)**		15 (40.50)	205 (54.80)	0.56 (0.28–1.12)	0.10
**Abdominal midline doming (yes), n (%)**		20 (54.10)	134 (36.40)	2.05 (1.04–4.06)	**0.04**
**DR severity** **n (%)**	**none, IRD < 2**	9 (24.30)	149 (40.50)	1.27 (0.95–1.69)	0.11
	**mild**, **IRD 2;< 3**	8 (21.60)	72 (19.60)		
	**moderate, IRD 3;<4**	12 (32.40)	75 (20.40)		
	**severe, IRD > 4**	8 (21.60)	72 (19.60)		
**Oxford scale** **Mean (SD)**		2.27(0.65)	2.28 (0.90)	0.99 (0.67–1.45)	0.96
**Reissing scale** **mean (SD)**		0.32 (1.11)	-0.53 (0.96)	1.25 (0.88–1.77)	0.21
**Correct activation of pelvic floor (yes), n (%)**		13 (35.10)	134 (35.80)	1.36 (0.69–2.70)	0.37

Based on the obtained prevalence of postpartum PGP, we could include up to 4 variables into the multivariable model [38]. The final model, in which we obtained statistically significant results for all included items, consisted of 3 variables. This multivariable regression analysis showed that the odds of having PGP shortly postpartum were higher in women with increased age (10% higher likelihood with every year of age), declaring PGP during pregnancy, and with higher values of Reissing scale. To enhance the interpretability, we then analyzed the Reissing scale in the following categories: hypotonus (range − 3 to -1) and increased muscle tone (range 1 to 3), with the reference value being normotonus (0). However, after this procedure, the Reissing scale became not statistically significant (Table 5). The calculated Nagelkerke R-square was 0.2.

**Table 5:** Multivariable analysis of factors associated with pelvic girdle pain (PGP) shortly postpartum (phase 1)

			OR (95% CI)	p-value
**Presence of PGP during pregnancy (yes)**			14.83 (4.340-48.721)	**< 0.0001**
**Age**			1.12 (1.009–1.214)	**0.032**
**Reissing scale**	**Reissing scale – not grouped**		1.43 (1.003–2.046)	**0.048**
	**Reissing scale when grouped**	**normal tone (0)**	reference	reference
		**decreased tone ** **(range − 3 to -1)**	0.53 (0.227–1.220)	0.134
		**increased tone ** **(range 1 to 3)**	0.37 (0.594–4.078)	0.368

## Discussion

Our study showed that nearly 10% of women were diagnosed with PGP during the first days postpartum, and almost 16% reported similar symptoms 6 weeks later. The mean pain intensity and functional limitations within the first days postpartum were moderate, with values corresponding to mild/low 6 weeks postpartum. The likelihood of experiencing PGP shortly after delivery increased with age and reporting PGP during pregnancy. The doming of the abdominal midline was significantly associated with PGP shortly postpartum only in the univariable analysis. The remaining variables related to diastasis recti or pelvic floor function were not associated with PGP shortly after delivery (24–72 h postpartum).

The reported prevalence of postpartum PGP around 12 weeks after delivery ranges from 3.4 to 43.0% [39–46] with the majority of studies [41–45] showing a higher prevalence than demonstrated in this research. This variation may be due to several reasons. Firstly, diverse diagnoses and terminology were used in the mentioned studies, which could lead to discrepancies in prevalence. In our research, postpartum PGP was defined as a pain that persisted postpartum or occurred within the first weeks after delivery [2]. However, in the study of Stomp van den Berg et al. [44] 25% of the 234 women who had PGP at 12 weeks postpartum had no PGP between 0 and 6 weeks after delivery. Secondly, cultural and ethnic factors can play a role in the processes related to pain perception [47]. Although PGP is prevalent worldwide, it is not recognized by health care systems in some countries. Our previous study has shown that PGP during pregnancy was more common in Norwegian than Polish women [6]. In Norway, PGP is one of the most common causes of sick leave among pregnant women [48]. In Poland, PGP is not commonly recognized, and the term ‘pelvic girdle pain’ is not widely used within health care services. Lower social awareness about this condition could lead to lower reporting. The possible role of ethnicity was noticed in another PGP study [49] indicating a more detailed investigation encompassing cultural and ethnic influences associated with PGP is needed.

We could observe similar discrepancies when analyzing the values related to pain intensity and functional limitations, possibly related to the same reasons as those mentioned above. For instance, in the study by Mukkanavar et al. [41] among Indian postpartum women, as many as 84.5% participants with PGP between the 3rd and 18th week after delivery rated their symptoms as greater than 60 mm on the VAS scale. Stomp van de Berg [44] reported median pain intensity 6 weeks postpartum at 4.3 of NRS scale. In the study of Dunn et al. [42] the mean pain intensity values measured on VAS scale were between 22.5 and 55, depending on the location of PGP and co-existing dysfunctions. Our results seem to be in line with those of Sakamoto et al. [50] who also measured functional limitations with PGQ. In the second day postpartum the mean values were oscillating around 47% (95%CI 40–54), while 4 weeks after − 19% (95%CI 12–25).

Our results showing higher PGP prevalence 6 weeks postpartum when compared to early postpartum period may seem contradictory to previous reports [37, 51]. However, it has to be noted that the cited studies followed women experiencing PGP already during pregnancy. The occurrence of pregnancy-related PGP may be delayed up to the first weeks postpartum [2]. By following all women (with and without pain), our study could capture those individuals that developed pain after the initial examination, 24-72 h postpartum. Additionally, early postpartum period is associated with more bed rest when compared to 6 weeks postpartum when PGP symptoms could be more noticeable and bothersome.

When it comes to factors associated with postpartum PGP, our results are in line with previous reports. A recent systematic review by Wiezer et al. [52] confirmed PGP during pregnancy as a risk factor for persistent postpartum pain. These findings suggest that asking women whether they have experienced PGP during pregnancy may help identify women at risk of persistent postpartum pain. The association between postpartum PGP and age has also been previously shown. Gausel et al. [46] reported age 30 and above as the risk factors for persistent postpartum pain. In European countries, primiparous women are becoming older. In Poland, the mean age of women having their first baby in 2019 was 27.4 and, although constantly increasing, is still one of the lowest in Europe [53]. Increasing maternal age may have several consequences. In accordance with previously mentioned studies, our results indicate that pregnant women who deliver past a certain age should receive special physiotherapy care.

In our study, the doming of the abdominal wall in the projection of linea alba was a statistically significant factor only in univariable analysis and there were no associations between the DRA severity (size of IRD) and the presence of PGP shortly postpartum. This is different when compared to our recently published matched-case control studies [54, 55]. However, in mentioned reports participants were matched according to age and parity, mode of delivery and time postpartum. This may suggest that although DRA features and postpartum PGP may co-exist, those associations are not straightforward and there are possibly other factors that may mediate this relationship.

Our study did not reveal any associations between pelvic floor function and PGP shortly postpartum, despite previous reports [17, 55]. This may be due to the timing of phase 1 of the study when the measurements were taken - some differences in the pelvic floor function may be too subtle to be detected using screening palpation examination in the early postpartum period. Our other hypothesis is that there are no differences in the pelvic floor muscle function between women with and without postpartum PGP shortly after delivery. They may be more visible with time, while pain persists and the adaptive changes in the pelvic floor occur, which could be supported by our other PGP study [55].

### Strengths and limitations

This was the first large-scale study conducted in Poland using the recommended guidelines for classifying and investigating postpartum PGP prevalence with the use of screening palpation examination of the pelvic floor and abdominal muscles. To our knowledge, this is also the first study in the central-eastern region in Europe. Considering that this region is inhabited mainly by Caucasian women with a similar physiognomy, our results may estimate postpartum PGP prevalence in this part of Europe.

The main limitation of this work is the high drop-out rate in phase 2 (“follow-up”). For this reason, the prevalence of PGP 6 weeks postpartum may be underestimated. Time constraints, lack of trust, and low awareness of clinical trials are the main barriers to participation in research projects [56]. Additionally, the first weeks after delivery are challenging for many women, and research obligations may not be their priority. High drop-out rates were also reported by another study investigating PGP 6 weeks postpartum via SMS where the response rate of 43% was recorded 6 weeks postpartum [37]. Another limitation could be caused by the assessment of PGP 6 weeks via self-reports. However, this method was used in previous PGP research [37, 42] and a study by Rejano-Campo et al. [57] showed that self-reported PGP was verified by specific clinical tests in nearly all cases.

Finally, we cannot exclude potential selection bias. Although we have made an effort to adopt random recruitment for phase 1 (shortly postpartum), we have included mainly highly educated women from only one center located in the capital city of Poland. This should be taken into account while inferring results from our sample to the general population.

### Implications

Obtained results with regard to other recently published matched case-control studies suggest that the relationship between PGP and DRA-related factors is multidimensional and not straightforward as previously suggested. Assessment of DRA-related factors seems not to be a crucial part of the screening for postpartum PGP but may be of greater importance when assessing individuals with postpartum PGP. Future research should further investigate the possible, multidimensional interactions between PGP and the whole abdominal wall complex (not restricted to only IRD as recommended by Delphi Consensus Study for the conservative management of pregnancy-related DRA [58]), and whether the DRA-related dysfunctions “only” co-exist with PGP or play a role in it. In that case, future studies focusing on creating adequate tension through the abdominal wall during PGP rehabilitation may be feasible. It should all be adjusted for psychosocial factors, which weren’t taken into account in our study. However, they are related to central pain mechanisms observed in individuals with persistent postpartum PGP and could be important factors filling the gaps in our current understanding of postpartum PGP [7, 59].

## Conclusion

The findings presented in this study suggest that every tenth Polish woman may experience PGP during the first days postpartum and every sixth can report similar symptoms 6 weeks later. The pain intensity and functional limitations tend to subside over time: from moderate pain intensity and functional limitation shortly postpartum to mild/low 6 weeks later. Nevertheless, postpartum PGP should not be ignored, especially in the context of the observed continued increase in chronic pain syndromes and their associated consequences. Older age, PGP during pregnancy, and doming of the abdominal midline at the level of linea alba were associated with the experience of PGP within the first days postpartum. Our study showed no association between pelvic floor function and PGP shortly postpartum. However, this may be due to the chosen methodology (assessment shortly postpartum).

## Data Availability

The raw data used to support the conclusions of this article are available from the respective corresponding author upon request.

## List of abbreviations

PGP: Pevic Girdle Pain
DRA: diastasis recti abdominis
IRD: inter-recti distance
ASLR: active straight leg raise
NRS: numerical rating scale
PGQ: Pelvic Girdle Questionnaire
MVC: maximal voluntary contraction
IRD: inter-recti distance
